# An analysis of a ‘community-driven’ reconstruction of the human metabolic network

**DOI:** 10.1007/s11306-013-0564-3

**Published:** 2013-07-12

**Authors:** Neil Swainston, Pedro Mendes, Douglas B. Kell

**Affiliations:** 1 School of Chemistry, The University of Manchester, Oxford Road, Manchester, M13 9PL UK; 2 Manchester Institute of Biotechnology, The University of Manchester, Princess Street, Manchester, M1 7DN UK; 3 School of Computer Science, The University of Manchester, Oxford Road, Manchester, M13 9PL UK; 4 Virginia Bioinformatics Institute, Virginia Tech, Washington St. 0477, Blacksburg, VA 24060 USA

**Keywords:** Metabolism, Modelling, Systems biology, Networks, Metabolic networks

## Abstract

Following a strategy similar to that used in baker’s yeast (Herrgård et al. Nat Biotechnol 26:1155–1160, 2008). A consensus yeast metabolic network obtained from a community approach to systems biology (Herrgård et al. 2008; Dobson et al. BMC Syst Biol 4:145, 2010). Further developments towards a genome-scale metabolic model of yeast (Dobson et al. 2010; Heavner et al. BMC Syst Biol 6:55, 2012). Yeast 5—an expanded reconstruction of the *Saccharomyces cerevisiae* metabolic network (Heavner et al. 2012) and in *Salmonella typhimurium* (Thiele et al. BMC Syst Biol 5:8, 2011). A community effort towards a knowledge-base and mathematical model of the human pathogen *Salmonella* *typhimurium LT2* (Thiele et al. 2011), a recent paper (Thiele et al. Nat Biotechnol 31:419–425, 2013). A community-driven global reconstruction of human metabolism (Thiele et al. 2013) described a much improved ‘community consensus’ reconstruction of the human metabolic network, called Recon 2, and the authors (that include the present ones) have made it freely available via a database at http://humanmetabolism.org/ and in SBML format at Biomodels (http://identifiers.org/biomodels.db/MODEL1109130000). This short analysis summarises the main findings, and suggests some approaches that will be able to exploit the availability of this model to advantage.

## Main findings of the Recon 2 paper

A highly curated consensus reconstruction of the human metabolic network, termed Recon 2, was recently released (Thiele et al. 2013). The development of the network followed a community ‘jamboree’ approach (Herrgård et al. 2008; Dobson et al. 2010; Heavner et al. 2012; Thiele and Palsson 2010), exploiting both genomic and literature data to expand upon existing reconstructions (Duarte et al. 2007; Ma et al. 2007; Gille et al. 2010) to produce a ‘basal’ network that contains 7,440 reactions, 5,063 metabolite pools and 2,626 unique metabolites. The reconstruction is extensively semantically annotated (Kell and Mendes 2008; Courtot et al. 2011), fully compliant with the MIRIAM standard (Le Novère et al. 2005), unambiguously identifying cellular compartments, metabolites, genes and enzymes with publicly available, external database terms (Krause et al. 2011). Thus, cellular compartments are annotated with Gene Ontology (GO) terms, while metabolites are annotated with terms from resources such as Chemical Entities of Biological Interest (ChEBI) (Hastings et al. 2013) as well as using IUPAC International Chemical Identifier (InChI http://www.iupac.org/home/publications/e-resources/inchi.html) terms (Coles et al. 2005) where possible. Reactions are curator-validated and annotated with PubMed literature references, standardized GO evidence codes, and a confidence scoring system ranging from 0 (no evidence) to 4 (biochemical evidence). Metabolic reactions were checked to ensure correct stoichiometry, (ir)reversibility, the correct assignment of gene association and enzyme rules, and mass and charge balancing. Appropriate transport reactions were also included and these followed the same level of annotation as reactions.

In contrast to existing resources such as KEGG (Kanehisa and Goto 2000) or the Human Metabolome Database (HMDB) (Wishart et al. 2007), Recon 2 acts as both a knowledgebase and a predictive model, amenable to constraint-based analysis approaches such as flux balance analysis (Orth et al. 2010). To demonstrate this utility, the Recon 2 authors focused on five analyses of immediate interest. First they defined a metabolic task as a nonzero flux through a reaction or through a pathway leading to the production of a metabolite B from a metabolite A; 354 such metabolic tasks were defined and all carried out successfully in silico. Secondly, they established whether known mutations producing ‘inborn errors of metabolism’ (IEMs) did have the predicted effect on biomarkers (54 reported biomarkers for 49 different IEMs, with an accuracy of 77 %; see also Shlomi et al. 2009). Thirdly, they showed that Recon 2 should predict a large fraction of metabolites that are excreted (the ‘metabolic footprint’ (Allen et al. 2003) or ‘exometabolome’ (Kell et al. 2005)). Fourthly, based on expression profiling data from the Human Protein Atlas (Uhlén et al. 2010), they generated 65 draft cell-type-specific models, and fifthly they found (notwithstanding the rather promiscuous behaviour of many drugs (Hopkins 2008, 2009; Kell et al. 2013)) that they could map 1,290 drugs to 308 enzyme and enzymatic complexes.

## Some known shortcomings of Recon 2

While Recon 2 represents the ‘state of the art’ of public human metabolic network reconstructions, it should be acknowledged that it does have some known shortcomings, including the fact that a number of known metabolites and reactions (including those involving unliganded iron (Hower et al. 2009; Kell 2009, 2010; Chifman et al. 2012; Funke et al. 2013)) have still to be included, and there are increasing numbers of ‘unexpected’ metabolite-protein reactions that are being discovered (Li et al. 2010; Li and Snyder 2011; Kell 2011; Kell et al. 2013). These are thus mainly ‘false negatives’ (Broadhurst and Kell 2006), and dealing with them is clearly one of the goals that will remain in any continuing curation process. It is recognised that the network reconstruction process is iterative (Reed and Palsson 2003), and the metabolomics and systems biology communities are encouraged to contribute to this ongoing effort. Following an approach that has been applied successfully in the generation of subsequent iterations of the yeast consensus model (Herrgård et al. 2008; Dobson et al. 2010; Heavner et al. 2012), suggested updates and amendments can be e-mailed to network.reconstruction@manchester.ac.uk.

## What the Recon 2 network will allow us to do or to do better

### General benefits of network models

**Fig. 1:**
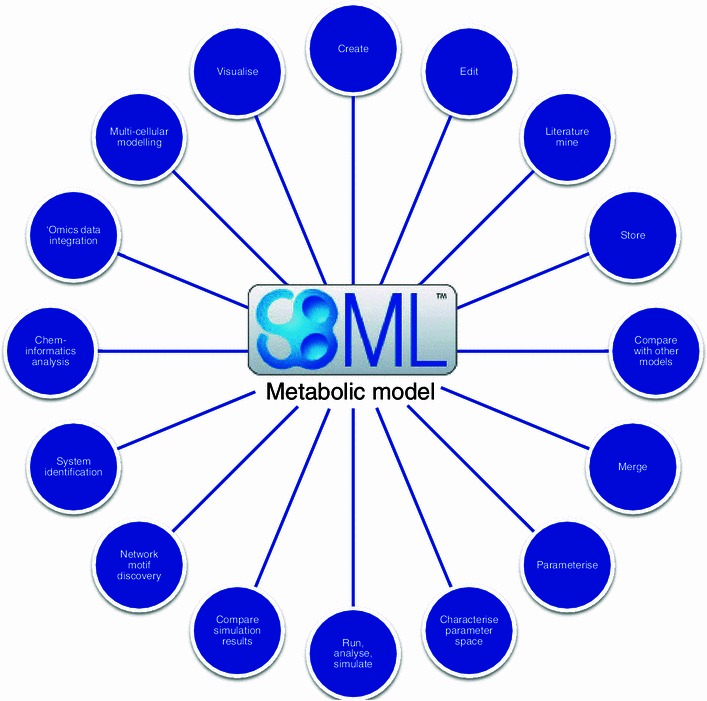
A summary of some of the intellectual areas in which we can create and exploit the contents of systems biology models as encoded in SBML

As discussed previously (Kell 2006a; b), the availability of a systems biology model of a metabolic network allows one to effect a variety of analyses, some of which are illustrated in Fig. 1. We here mention just a few that are likely to be of most interest to the metabolomics community.

### Improved predictions of metabolic fluxes, including in biotechnology

Given the topology of a network, and the stoichiometric and thermodynamic constraints under which metabolic networks must operate (Palsson 2006; Kell 2006a; b), it is possible to use generalised kinetics to predict metabolic fluxes (Liebermeister and Klipp 2006; Smallbone et al. 2007; Smallbone and Simeonidis 2008; Smallbone et al. 2010). The accuracy of these predictions can of course be enhanced by the use of known kinetic rate equations (Li et al. 2010), and even by expression profiles alone (Lee et al. 2012). Such an approach has been applied, exploiting both transcriptomics and fluxomics data, to constrain models derived from a precursor of Recon 2 in order to elucidate and validate new drug targets in renal-cell cancer (Frezza et al. 2011).

The use of network biology in predicting fluxes (and how to change them), as well as in parameter optimisation (Mendes and Kell 1998; Moles et al. 2003; Adams et al. 2013), has enjoyed particular success in biotechnology where it is usually the fluxes to external products that are of interest (Park et al. 2007; Lee et al. 2012; Park et al. 2010; Becker et al. 2011). In this area, the ongoing development of a systems biology toolkit for Chinese Hamster Ovary (CHO) cells, which will be increasingly utilised for biotechnological production of pharmaceutical proteins (Kildegaard et al. 2013), will be aided by the development of Recon 2, which can act as a template for development of a detailed metabolic reconstruction of CHO. Additionally, one may anticipate the importance of predictions of metabolic fluxes in understanding nutrition and regulation in health and disease.

### Understanding and incorporating knowledge of drugs that use known transporters

As part of the need to incorporate ‘new’ proteins and their interactions with small molecules, one particular feature that has become increasingly apparent in recent years is that pharmaceutical drugs do not normally cross membranes ‘passively’ through any phospholipid bilayer portions that they may contain, but hitchhike on the carriers that participate in the transmembrane transport of intermediary metabolites (Al-Awqati 1999; Dobson and Kell 2008; Dobson et al. 2009; Dobson et al. 2009; Kell and Dobson 2009; Giacomini et al. 2010; Burckhardt and Burckhardt 2011; Kell et al. 2011; Lanthaler et al. 2011; DeGorter et al. 2012; Kell and Goodacre 2013). It is likely that these kinds of issues contribute significantly to the dreadful attrition rates still seen in drug development (van der Greef and McBurney 2005; Kola and Landis 2004; Kola 2008; Empfield and Leeson 2010; Leeson and Empfield 2010; Kwong et al. 2011). The availability of Recon 2 and its tissue-specific versions will now make it much easier to correlate drug disposition with transporter expression, and thereby determine (with suitable machine learning analyses (Kell et al. 2001)) the roles of the different transporters in effecting the cellular uptake and efflux of particular drugs. Incorporating this kind of knowledge into subsequent iterations of Recon 2 is an urgent priority.

### Other approaches to mining the metabolic network

**Fig. 2:**
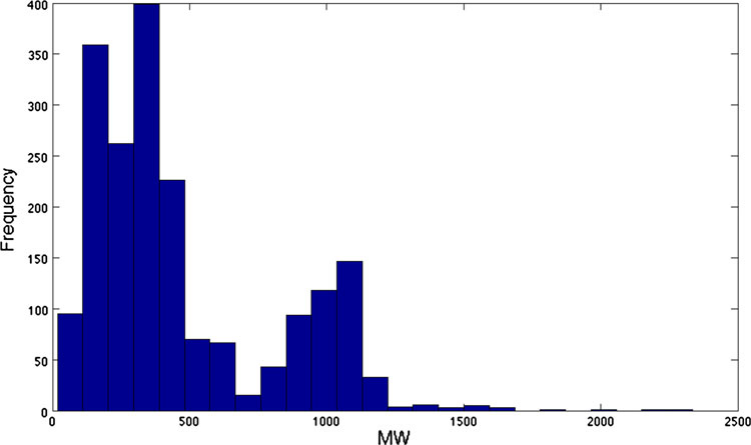
An assessment of the distribution of molecular masses of the metabolites in Recon 2

An important recognition (Herrgård et al. 2008), continued in Recon 2, was that of the utility of the methods of cheminformatics (Gasteiger 2003) in providing chemically accurate and database-independent descriptions of the structures of metabolites that allowed models (such as those encoded in SBML (Hucka et al. 2003; Hucka et al. 2004)) to be interrogated computationally. In a similar vein, there is an increasing trend towards automated reasoning about the content of scientific papers from a systems biology point of view (Hakenberg et al. 2004; King et al. 2005; Ananiadou et al. 2006; Kell and Mendes 2008; Ananiadou et al. 2010; Ray et al. 2010; Miwa et al. 2012, 2013), including about their metabolomes (Knox et al. 2007; Attwood et al. 2009; Attwood et al. 2010; Nobata et al. 2011; Zhou et al. 2012; Hastings et al. 2013). The availability of Recon 2 will allow one to ask questions such as, “how many metabolites with a given substructure are present in the network?” or “which metabolites are common (or different) between these two networks?” or to plot out the distributions of various kinds of properties that may be of interest (Dobson et al. 2009); one such plot, simply showing the distribution of molecular masses, is given in Fig. 2.

### Comparison with the experimental metabolome

Analysis of the metabolome as encoded in biochemical networks also leads one to recognise the importance of comparing systems biology models with the experimental metabolome (i.e. the concentrations of small molecules) measured in different circumstances (‘comparative metabolomics’, Raamsdonk et al. 2001; Levandi et al. 2008). As with Recon 2, the experimental metabolome of, for example, human serum consists of several thousands of reproducibly detectable metabolites (O’Hagan et al. 2005; 2007; Begley et al. 2009; Zelena et al. 2009). While some of these experimentally observed metabolites will certainly originate from nutrients or the gut microbiome (Goodacre 2007; Li et al. 2008; Wikoff et al. 2009; Zhao and Shen 2010; Wang et al. 2011; Bennett et al. 2013; Collino et al. 2013; Heinken et al. 2013), Recon 2 allows one to set down those that are at least encoded in the human genome sequence, and compare these with the contents of the various metabolome databases (Brown et al. 2009; Kamp et al. 2012; van Ravenzwaay et al. 2012; Sawada et al. 2012; Steinbeck et al. 2012; Tautenhahn et al. 2012; Wishart 2012; Zhou et al. 2012; Guo et al. 2013; Hastings et al. 2013; Haug et al. 2013; Li et al. 2013; Salek et al. 2013; Sakurai et al. 2013; Wishart et al. 2013). Data standards such as SBRML (Dada et al. 2010) allow a straightforward comparison of network models with experimental data encoded in a compatible format.

### The importance of semantic annotation

Many of the application areas described above are dependent upon the semantic awareness of Recon 2, and the incorporation of thousands of unique, persistent, unambiguous semantic annotations that allow for software-driven analyses of the knowledgebase and derived models. By representing both the network and its semantic information using community-driven standards such as SBML (Hucka et al. 2003, 2004) and MIRIAM (Le Novère et al. 2005), software producers are able to develop against a given standard, decoupling the network model from the techniques used in its various more specialized analyses.

There is increasing community interest in the development of tissue- and condition-specific models, and this task is dependent upon the integration of large-scale ‘omics data. Methods to perform such integration are many-fold and are in constant development (Mo et al. 2009; Jerby et al. 2010; Wang et al. 2012), but all are reliant on automated approaches, given that the size of the datasets involved renders manual integration impossible. Recon 2 is annotated with third-party identifiers across numerous scales, from genomics through to transcriptomics, proteomics and metabolomics, all of which can be mapped to appropriate web services, allowing for their automated interpretation (Swainston and Mendes 2009) and integration of multi-omics data (Li et al. 2008, 2008; Hyduke et al. 2013). Additionally, the definition of metabolites in structural terms permits the exploitation of cheminformatics tools such as the Chemistry Development Toolkit (Steinbeck et al. 2003) and Open Babel that allow one to translate the various encodings or mappings of chemical structures (O’Boyle et al. 2011).

Furthermore, the specification of metabolites, enzymes and reactions in unambiguous terms facilitates the development of knowledgebases and models of related organisms, through automated or semi-automated means (Henry et al. 2010; Swainston et al. 2011; Agren et al. 2013). Recon 2 can therefore act as a template for the development of metabolic reconstructions of related model organisms, facilitating comparative studies and simulation of metabolism between human and other model systems (Sigurdsson et al. 2010).

## Concluding remarks

The availability of Recon 2 allows a great many computational analyses to be performed. We have purposely rehearsed these at a rather general level, since particular analyses, that might be relevant to particular diseases, for instance, are simply implementations of the more general approaches. One new approach that will depend on the existence of such a network as a necessary resource is personalised medicine (Hood and Flores 2012). There one will develop models of metabolism calibrated for each specific individual, in large part using metabolomics methods, to be used as bases for diagnostics and decisions on course of treatment. Recon 2 is a very significant step towards such a map, where such measurements have to be anchored for various types of modelling that will underpin personalised treatment decisions.

The development of tissue- and condition-specific models has been demonstrated with Recon 2 and its predecessors (Jerby et al. 2010; Frezza et al. 2011; Wang et al. 2012). As subsequent iterations of Recon 2 develop, it is hoped that the scope of the knowledgebase, and the predictive power of derived models, will increase to keep pace with advancements in the community knowledge of human metabolism, many of which will be driven by the discipline of metabolomics.
